# Supplementary material for: Future Deployment and Flexibility of Distributed Energy Resources in the Distribution Grids of Switzerland
Source: Sci Data. 2025 Aug 26;12:1491. doi: 10.1038/s41597-025-05830-y (PMC12381229; doi:10.1038/s41597-025-05830-y)
Supplement: Supplementary file 1 — Supplementary Information [file 41597_2025_5830_MOESM1_ESM.pdf]

Supplementary Information
Future Deployment and Flexibility of Distributed Energy Resources in the Distribution Grids of Switzerland

Lorenzo Zapparoli[*1], Alfredo Oneto[*1], María Parajeles Herrera[2], Blazhe Gjorgiev[1], Gabriela Hug[2], and Giovanni Sansavini[†1]

[1]Reliability and Risk Engineering Lab, Institute of Energy and Process Engineering, Department of Mechanical and Process Engineering, ETH Zürich, Switzerland
[2]Power Systems Lab, Institute for Power Systems & High Voltage Technology, Department of Information Technology and Electrical Engineering, ETH Zürich, Switzerland

[*]These authors contributed equally.
[†]Corresponding author: sansavig@ethz.ch

Description of the distribution grids by Canton and for Switzerland

Table 1: Description of the distribution grids by Canton (non-bold codes) and for Switzerland (CH). For each coded area, the number of grids, load nodes, average peak power, and standard deviation of peak power are provided for medium- and low-voltage levels. In this regard, load nodes refer to electrical nodes in the distribution grid that contain non-controllable loads, except for the transformers.

**Code**	**Medium-voltage grids**				**Low-voltage grids**			
	**Number**	**Load nodes**	**Avg.** $\overline{P}$ **(MW)**	**Std.** $\overline{P}$ **(MW)**	**Number**	**Load nodes**	**Avg.** $\overline{P}$ **(kW)**	**Std.** $\overline{P}$ **(kW)**
AG	58	3,141	13.02	5.67	2,112	156,484	312.32	154.05
AI	1	154	20.41	0	186	5,674	142.41	75.64
AR	8	317	8.73	6.1	290	16,721	257.63	165.24
BE	107	6,142	12.29	5.65	4,767	272,916	280.62	166.53
BL	22	1,378	16.1	4.65	773	61,257	370.21	145.62
BS	19	813	15.92	4.1	447	25,392	433.35	112.15
FR	32	1,763	11.73	5.74	1,336	74,678	271.76	154.83
GE	37	2,112	14.51	4.86	1,007	45,193	404.44	132.29
GL	11	715	14.24	6.24	522	17,658	284.64	172.68
GR	40	2,321	9.54	6.27	2,150	78,966	201.08	126.25
JU	13	549	8.28	3.73	454	24,979	234.93	147.42
LU	43	2,439	11.7	5.29	1,799	80,805	252.4	156.8
NE	18	956	12.16	5.37	607	32,348	309.2	174.89
NW	5	302	10.18	6.6	279	9,300	235.18	148.11
OW	8	399	8.08	4.41	362	12,280	191.19	110.89
SG	54	3,123	13.68	5.3	2,036	127,847	300.67	168.86
SH	6	294	11.73	4.16	278	20,053	334.09	154.19
SO	20	1,280	15.33	5.51	922	74,568	314.75	152.67
SZ	16	935	12.41	6.56	696	35,655	299.11	170.33
TG	28	1,623	12.1	5.19	1,192	74,855	253.09	148.19
TI	49	2,661	12.75	5.61	1,994	96,183	335.9	162.91
UR	4	244	13.91	8.24	204	10,404	314.68	174.24
VD	71	4,020	12.91	6.14	2,721	139,528	321.7	165.55
VS	80	4,899	12.66	4.98	3,706	114,664	283.71	158.84
ZG	14	787	13.67	6.68	385	17,088	380.84	164.72
ZH	115	6,283	13.82	5.15	3,695	236,848	384.65	141.39
**CH**	**879**	**49,650**	**12.81**	**5.62**	**34,920**	**1,862,344**	**302.02**	**164.08**

Detailed data repository description

Table 2: Data repository and its description.

Output files			
**Archive folder**	**Year folder**	**Files**	**Description**
01_PV	2030<br>2040<br>2050	LV_generation.csv	**Columns:** *column 1* - LV_grid (identifier of the low-voltage grid),<br>*column 2* - LV_osmid (identifier of the low-voltage node),<br>*columns 3-290* - 288 time-steps of 1 hour (1 day with 1 hour resolution for each month of the year).<br>**Rows:** 481,318 rows for 2030,<br>758,118 rows for 2040,<br>1,427,096 rows for 2050 (low-voltage nodes with PV installation).<br>**Comment:** PV active power generation profiles for low-voltage nodes (kW).
		LV_P_installed.csv	**Columns:** *column 1* - LV_grid (identifier of the low-voltage grid),<br>*column 2* - LV_osmid (identifier of the low-voltage node),<br>*column 3* - P_installed_kW (nominal installed PV capacity in kWp).<br>**Rows:** 481,318 rows for 2030,<br>758,118 rows for 2040,<br>1,427,096 rows for 2050 (low-voltage nodes with PV installation).<br>**Comment:** Nominal installed PV capacity in kWp for the low-voltage nodes.
		LV_std.csv	**Columns:** *column 1* - LV_grid (identifier of the low-voltage grid),<br>*column 2* - LV_osmid (identifier of the low-voltage node),<br>*columns 3-290* - 288 time-steps of 1 hour (1 day with 1 hour resolution for each month of the year).<br>**Rows:** 481,318 rows for 2030,<br>758,118 rows for 2040,<br>1,427,096 rows for 2050 (low-voltage nodes with PV installation).<br>**Comment:** Standard deviation for PV active power generation profiles for low-voltage nodes (kW).
		MV_generation.csv	**Columns:** *column 1* - MV_grid (identifier of the medium-voltage grid),<br>*column 2* - MV_osmid (identifier of the medium-voltage node),<br>*columns 3-290* - 288 time-steps of 1 hour (1 day with 1 hour resolution for each month of the year).<br>**Rows:** 11,551 rows for 2030,<br>14,759 rows for 2040,<br>19,452 rows for 2050 (medium-voltage nodes with PV installation).<br>**Comment:** PV active power generation profiles for medium-voltage nodes (kW).

		MV_P_installed.csv	**Columns:** *column 1* - MV_grid (identifier of the medium-voltage grid),<br>*column 2* - MV_osmid (identifier of the medium-voltage node),<br>*column 3* - P_installed_kW (nominal installed PV capacity in kWp).<br>**Rows:** 11,551 rows for 2030,<br>14,759 rows for 2040,<br>19,452 rows for 2050 (medium-voltage nodes with PV installation).<br>**Comment:** Nominal installed PV capacity in kWp for medium-voltage nodes.
		MV_std.csv	**Columns:** *column 1* - MV_grid (identifier of the medium-voltage grid),<br>*column 2* - MV_osmid (identifier of the medium-voltage node),<br>*columns 3-290* - 288 time-steps of 1 hour (1 day with 1 hour resolution for each month of the year).<br>**Rows:** 11,551 rows for 2030,<br>14,759 rows for 2040,<br>19,452 rows for 2050 (medium-voltage nodes with PV installation).<br>**Comment:** Standard deviation PV active power generation profiles for medium-voltage nodes (kW).
02_BESS	2030<br>2040<br>2050	BESS_allocation_LV.csv	**Columns:** *column 1* - LV_grid (identifier of the low-voltage grid),<br>*column 2* - LV_osmid (identifier of the low-voltage node),<br>*column 3* - Battery_capacity_kWh (storage capacity of the BESS in kWh),<br>*column 4* - Nominal_power_kW (rated power of the BESS in kW),<br>*column 5* - Charging_efficiency (charging efficiency of the BESS),<br>*column 6* - Discharging_efficiency (discharging efficiency of the BESS).<br>**Rows:** 151,034 rows for 2030,<br>384,287 rows for 2040,<br>998,961 rows for 2050 (low-voltage nodes with BESS co-installed with PV).<br>**Comment:** The parameters of the BESS installed at the low-voltage nodes.
		BESS_allocation_MV.csv	**Columns:** *column 1* - MV_grid (identifier of the medium-voltage grid),<br>*column 2* - MV_osmid (identifier of the medium-voltage node),<br>*column 3* - Battery_capacity_kWh (storage capacity of the BESS in kWh),<br>*column 4* - Nominal_power_kW (rated power of the BESS in kW),<br>*column 5* - Charging_efficiency (charging efficiency of the BESS),<br>*column 6* - Discharging_efficiency (discharging efficiency of the BESS).<br>**Rows:** 3,625 rows for 2030,<br>7,449 rows for 2040,<br>13,726 rows for 2050 (medium-voltage nodes with BESS co-installed with PV).<br>**Comment:** The parameters of the BESS installed at the medium-voltage nodes.

03_HP	2030<br>2040<br>2050	LV_heat_pump_allocation.csv	**Columns:** *column 1* - LV_grid (identifier of the low-voltage grid),<br>*column 2* - LV_osmid (identifier of the low-voltage node),<br>*columns 3* - Nominal_power_kW (nominal electrical power of the heat pumps connected to the node),<br>*column 4* - Thermal_capacitance_KWh/K (thermal capacitance of the served buildings),<br>*column 5* - Thermal_conductivity_kW/K (thermal conductivity of the served buildings),<br>*columns 6* - COP (fixed coefficient of performance),<br>*column 7* - COP_0 (constant term of the variable coefficient of performance),<br>*columns 8* - COP_1 (first-order term of the variable coefficient of performance),<br>*columns 9* - COP_2 (second-order term of the variable coefficient of performance),<br>*columns 10* - Temperature_profile_name (identifier pointing to the temperature profile in Temperature_profiles.csv).<br>**Rows:** 630,171 rows for 2030,<br>876,594 rows for 2040,<br>1,065,403 rows for 2050 (low-voltage nodes with HP installation).<br>**Comment:** Nominal installed electrical HP capacity in kW for low-voltage nodes, the thermal capacitance/conductivity of the served buildings per node, the HPs coefficient of performance, and the outdoor temperature profile name identifier.

		MV_heat_pump_allocation.csv	**Columns:** *column 1* - MV_grid (identifier of the medium-voltage grid),<br>*column 2* - MV_osmid (identifier of the medium-voltage node),<br>*column 3* - Nominal_power_kW (nominal electrical power of the heat pumps connected to the node),<br>*column 4* - Thermal_capacitance_KWh/K (thermal capacitance of the served buildings),<br>*column 5* - Thermal_conductivity_kW/K (thermal conductivity of the served buildings),<br>*columns 6* - COP (fixed coefficient of performance),<br>*column 7* - COP_0 (constant term of the variable coefficient of performance),<br>*columns 8* - COP_1 (first-order term of the variable coefficient of performance),<br>*columns 9* - COP_2 (second-order term of the variable coefficient of performance),<br>*column 10* - Temperature_profile_name (identifier pointing to the temperature profile in Temperature_profiles.csv).<br>**Rows:** 11,202 rows for 2030,<br>13,481 rows for 2040,<br>15,429 rows for 2050 (medium-voltage nodes with HP installation).<br>**Comment:** Nominal installed electrical HP capacity in kW for medium-voltage nodes, the thermal capacitance/conductivity of the served buildings per node, the HPs coefficient of performance, and the outdoor temperature profile name identifier.
		Temperature_profiles.csv	**Columns:** *column 1* - outdoor temperature profile identifier,<br>*columns 2-8,761* - 8,760 time-steps of 1 hour (365 days with 1 hour resolution).<br>**Rows:** 440 rows (one for each outdoor temperature yearly profile)<br>**Comment:** Yearly, hourly resolved, ambient temperature profile (in degrees Celsius) from weather stations.
04_EV	2030<br>2040<br>2050	EV_power_profiles_LV.csv	**Columns:** *column 1* - BFS_municipality_code (identifier for the municipality),<br>*column 2* - Profile_type (indicates the type of profile, i.e., Upper, Base, or Lower),<br>*column 3-8,762* - 8,760 time-steps of 1 hour (365 days with 1 hour resolution).<br>**Rows:** 6,444 rows (2,148 municipalities × 3 profile types).<br>**Comment:** The base profile represents the uncontrolled charging demand, while the Upper and Lower bounds define the maximum and minimum power levels, respectively (kW).

		EV_flexible_energy_profiles_LV.csv	**Columns:** *column 1* - BFS_municipality_code (identifier for the municipality),<br>*columns 2-366* - 365 time-steps of 1 day.<br>**Rows:** 2148 rows (one for each municipality identifier).<br>**Comment:** Maximum flexible energy that can be shifted per day from the base power charging profile (kWh).
		EV_allocation_LV.csv	**Columns:** *column 1* - LV_grid (identifier of the low-voltage grid, where the number before the dash corresponds to the BFS code of the municipality, e.g., 852-2_1_2 belongs to municipality 852),<br>*column 2* - LV_osmid (identifier of the low-voltage node),<br>*column 3* - EV_share (share factor indicating the fraction of the municipality-level profile assigned).<br>**Rows:** 2,525,530 (low-voltage nodes in the country).<br>**Comment:** Distributes the municipality-level EV profiles to low-voltage nodes (the shares are unitless).
05_Demand	2030<br>2040<br>2050	LV_basicload_shares.csv	**Columns:** *column 1* - LV_grid (identifier of the low-voltage grid),<br>*column 2* - LV_osmid (identifier of the low-voltage node),<br>*column 3* - Commercial_demand_share (share of commercial demand at the low-voltage node),<br>*column 4* - Residential_demand_share (share of residential demand at the low-voltage node).<br>**Rows:** 2,525,530 (low-voltage nodes in the country).<br>**Comment:** Share of commercial and residential demand for each low-voltage node.
		Commercial_profiles.csv	**Columns:** *column 1* - BFS_municipality_code (identifier for the municipality),<br>*columns 2-8761* - 8,760 time-steps of 1 hour (365 days with 1 hour resolution).<br>**Rows:** 2,148 rows (one for each municipality identifier).<br>**Comment:** Hourly max-normalized load profile for commercial demand at the municipality level, identified by the BFS municipality code.
		Residential_profiles.csv	**Columns:** *column 1* - BFS_municipality_code (identifier for the municipality),<br>*columns 2-8761* - 8,760 time-steps of 1 day (365 days with 1 hour resolution).<br>**Rows:** 2,148 rows (one for each municipality identifier).<br>**Comment:** Hourly max-normalized load profile for residential demand at the municipality level, identified by the BFS municipality code.

		MV_load_profile.csv	**Columns:** *column 1-8,760* - 8,760 time-steps of 1 hour (365 days with 1 hour resolution)<br>**Rows:** *row 1* - Power_pu (max-normalized MV demand).<br>**Comment:** Representative hourly max-normalized non-controllable load profile for medium-voltage nodes.
**Additional folders**			
Archive folder		Description	
06_Grids		In this repository, only the grids for the integrated medium-low voltage system 459_0 are provided. To access all available grids, refer to the original grid repository and replace the LV and MV zip files accordingly. For further details on the grid repository data, refer to the article. The folder contains the following files:<br>*LV.zip:* This .zip archive contains all the low-voltage power distribution grids data, such as grid topology, branch flow limits, line impedance, and nodal peak powers.<br>*MV.zip:* This .zip archive contains all the medium-voltage power distribution grids data, such as grid topology, branch flow limits, line impedance, and nodal peak powers.<br>*dict_folder.json:* This file contains a dictionary that maps the BFS municipality number, the number before the dash (-) of the low-voltage grid code, to the corresponding subfolder in the LV.zip archive. This file is used to access the low-voltage grid data.	
07_Complementary_data		This folder contains complementary data, which are not needed to use the DERs dataset but may be of use for its interpretation and modification. It contains a file with municipalities information.<br>*Municipalities_2022_01_crs2056.geojson:* This GeoJSON file contains the municipal boundaries of Switzerland as part of the swissBOUNDARIES 3D dataset, based on EPSG:2056 coordinate reference system.<br>**Columns:** This file contains 24 columns. The most relevant for the purpose of this dataset are listed below.<br>*'BFS_NUMMER'* - Identifier for the municipality.<br>*'NAME'* - Name of the municipality.<br>*'ICC'* - Country code: CH for Switzerland, LI for the Principality of Liechtenstein, DE for Germany, and IT for Italy.<br>*'EINWOHNERZ'* - Population of the municipality as of 2022.<br>*'geometry'* - Geometry of the municipality, in the EPSG:2056 projected coordinate system.<br>**Rows:** 2,174 rows, including 2,148 inhabited Swiss municipalities and 13 Swiss municipalities with no reported permanent residents. The remaining 13 municipalities belong to the Principality of Liechtenstein or are exclaves of neighboring countries.<br>**Comment:** The geometry and attributes of the municipalities in Switzerland, the Principality of Liechtenstein, and exclaves from neighboring countries. It reflects the municipal boundaries as of January 2022.	
**Additional files**			
File		Description	
08_Data_loader.py		This Python script provides an example of how to load distributed energy resources (DERs) data for low-voltage and medium-voltage grids for a specified simulation year and time interval. The script is designed to facilitate the loading and processing of the DER data present in the dataset.	

Nodal Power Distribution of Distributed Energy Resources

Figure 1 summarizes the distribution of nodal power allocation for PVs, BESSs, and HPs at low-voltage and medium-voltage levels, projected for 2050. Panel (a) shows the low-voltage distributions, and panel (b) shows the medium-voltage distributions.

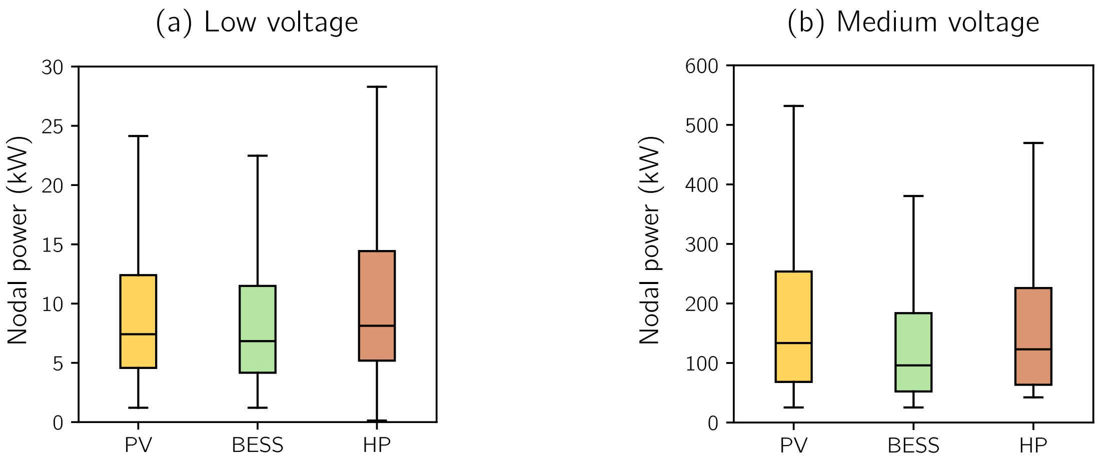

Figure 1: The panels show the allocated nodal power distribution of PVs, BESSs, and HPs in the Swiss distribution grids for 2050. Panel (a) presents box plots of the installed PV power, BESS charging/discharging power, and HP electrical power at low-voltage nodes; panel (b) shows the corresponding distributions for medium-voltage nodes.

Aggregated profiles in Switzerland

At both medium- and low-voltage levels, aggregated time profiles for Switzerland are obtained for PV, HP, EV, and non-controllable loads. Future projections are included for the years 2030, 2040, and 2050, as shown in Figure 2. Energy consumption and generation profiles are provided for an arbitrary day (March 11) and each month of the year, in addition to daily variability for each data category included across the respective years. Panels (a), (b), and (c) correspond to 2030; (d), (e), and (f) to 2040; and (g), (h), and (i) to 2050.

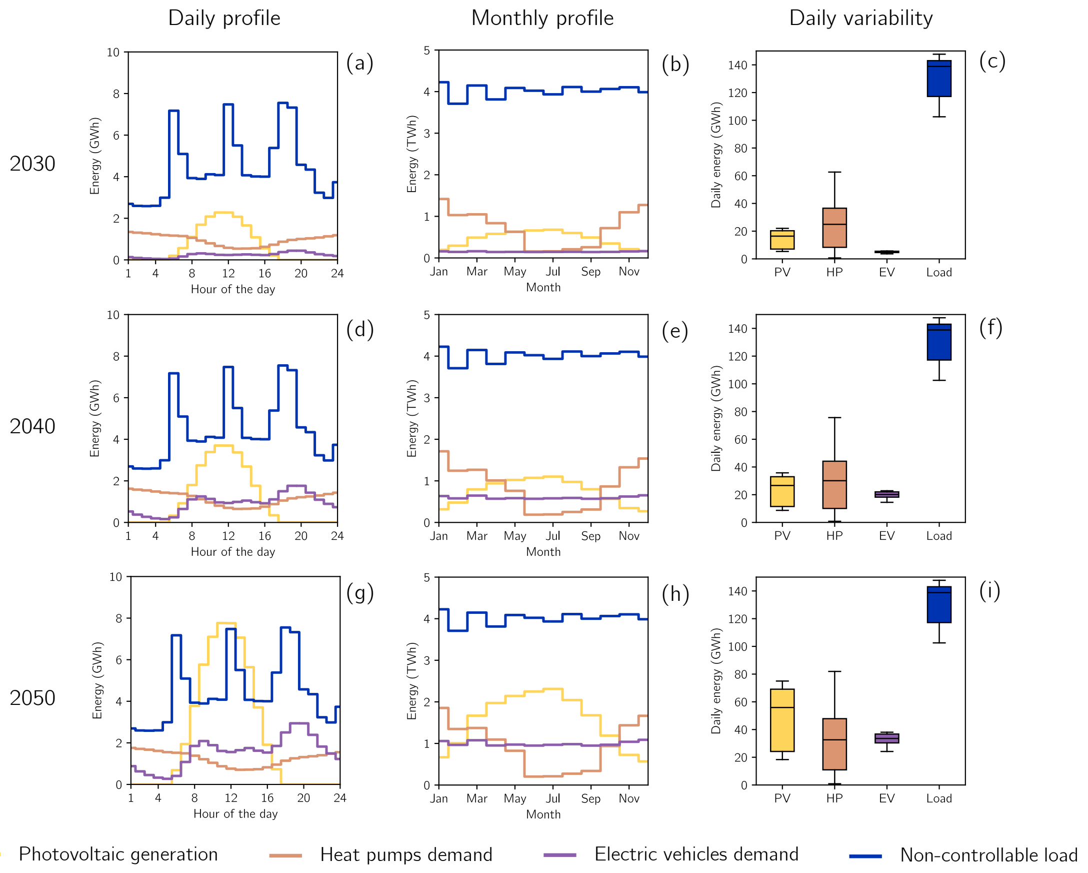

Figure 2: Daily profiles, monthly profiles, and daily variability graphs are shown for the entire dataset in Switzerland. Panels (a), (b), and (c) correspond to projections for 2030; (d), (e), and (f) to 2040; and (g), (h), and (i) to 2050.

Correlation of Distributed Energy Resource Deployment with Population

More populated areas are expected to have a higher number of distributed energy resources. Therefore, a realistic geographical distribution in the dataset should reflect this pattern. In Figure 3, the projected deployment of each distributed energy resource category for 2050 is compared with the current population of Swiss municipalities. Panel (a) presents the installed PV power, (b) the co-located BESS charging/discharging power, (c) the electrical HP power, and (d) the peak EV base charging power, each matched with the corresponding municipal population. The graphs confirm a clear trend between distributed energy resource power and population.

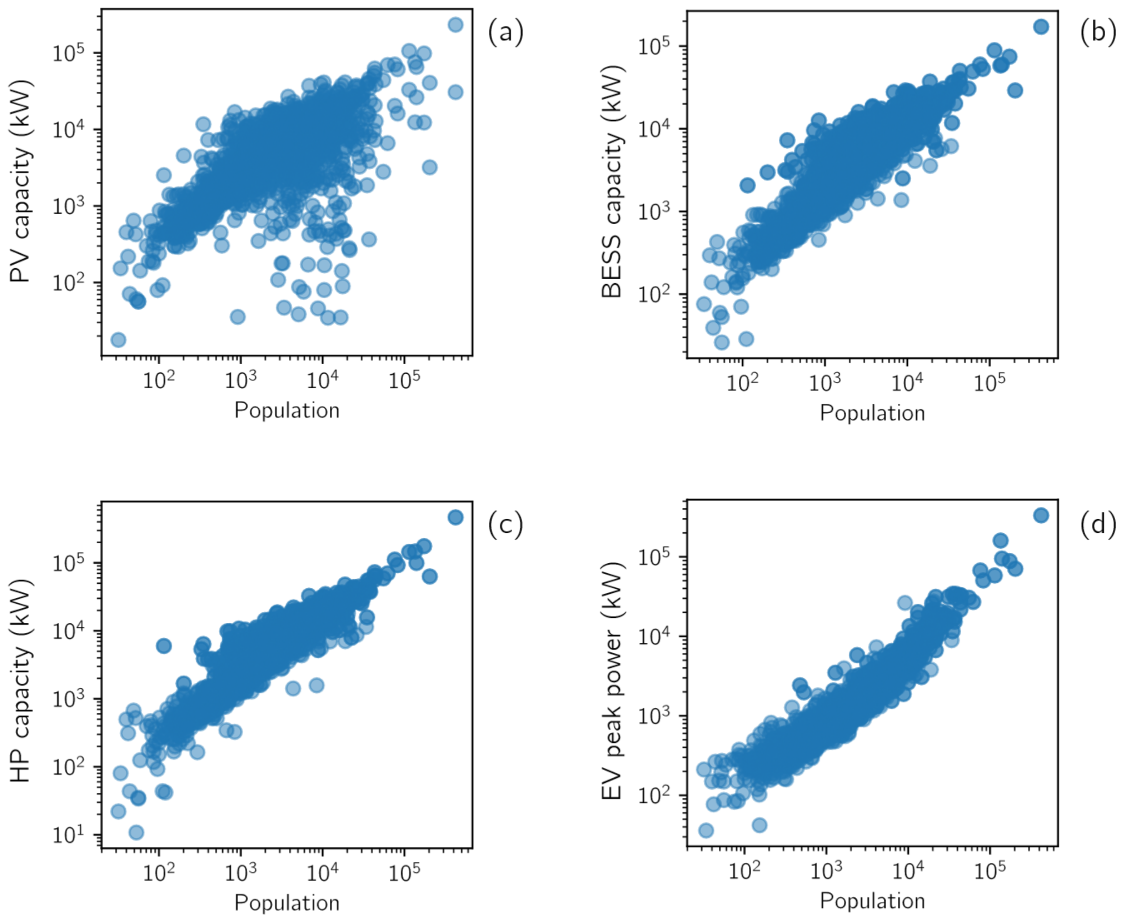

Figure 3: The deployment of each distributed energy resource in 2050 is shown against the current population of Swiss municipalities. Panel (a) presents the installed PV power, (b) the co-located BESS charging/discharging power, (c) the electrical HP power, and (d) the peak EV base charging power.
